# Multiview Consensus Graph Learning for lncRNA–Disease Association Prediction

**DOI:** 10.3389/fgene.2020.00089

**Published:** 2020-02-21

**Authors:** Haojiang Tan, Quanmeng Sun, Guanghui Li, Qiu Xiao, Pingjian Ding, Jiawei Luo, Cheng Liang

**Affiliations:** ^1^ School of Information Science and Engineering, Shandong Normal University, Jinan, China; ^2^ School of Information Engineering, East China Jiaotong University, Nanchang, China; ^3^ College of Information Science and Engineering, Hunan Normal University, Changsha, China; ^4^ School of Computer Science, University of South China, Hengyang, China; ^5^ College of Computer Science and Electronic Engineering, Hunan University, Changsha, China

**Keywords:** lncRNA–disease association, multiple similarity matrices, consensus graph learning, multi-label learning, survival analysis

## Abstract

Long noncoding RNAs (lncRNAs) are a class of noncoding RNA molecules longer than 200 nucleotides. Recent studies have uncovered their functional roles in diverse cellular processes and tumorigenesis. Therefore, identifying novel disease-related lncRNAs might deepen our understanding of disease etiology. However, due to the relatively small number of verified associations between lncRNAs and diseases, it remains a challenging task to reliably and effectively predict the associated lncRNAs for given diseases. In this paper, we propose a novel multiview consensus graph learning method to infer potential disease-related lncRNAs. Specifically, we first construct a set of similarity matrices for lncRNAs and diseases by taking advantage of the known associations. We then iteratively learn a consensus graph from the multiple input matrices and simultaneously optimize the predicted association probability based on a multi-label learning framework. To convey the utility of our method, three state-of-the-art methods are compared with our method on three widely used datasets. The experiment results illustrate that our method could obtain the best prediction performance under different cross validation schemes. The case study analysis implemented for uterine cervical neoplasms further confirmed the utility of our method in identifying lncRNAs as potential prognostic biomarkers in practice.

## Introduction

With the completion of ENCODE project, researchers have found that only 2% of genes in the human genome encode proteins, while approximately 75% of the human genome is involved in the process of primary transcripts (Djebali et al., 2012; Li and Chang, 2014; Zhang et al., 2018b). The discovery of extensive transcription of large RNA transcripts which do not code for proteins, termed long noncoding RNAs (lncRNAs), provides a new perspective in understanding the centrality of RNA in gene regulation (Rinn and Chang, 2012). Evidences have shown that lncRNAs are key regulators for many cellular functions, including splicing, gene regulation, and hormone-like activity (Gao et al., 2019a; Mongelli et al., 2019). Moreover, the dysregulation of lncRNAs has been proved to be closely related with various human diseases, such as types of cancer, neurological as well as cardiovascular diseases (Feng et al., 2018; Zhang et al., 2019b). Consequently, identifying potential disease-related lncRNAs is of great importance and might shed new light on the understanding of the pathogenesis of complex diseases.

As a powerful complementary tool for biological and clinical experiments, many computational approaches have been developed to effectively predict the lncRNA-disease associations (Zou et al., 2016; Chen et al., 2017; Zhang et al., 2018c; Gong et al., 2019; Yue et al., 2019). Under the assumption that similar diseases are more likely to be associated with functionally similar lncRNAs, Chen et al. proposed Laplacian regularized least squares for lncRNA-disease association in terms of a semi-supervised learning framework(Chen and Yan, 2013). Liu et al. combined the gene expression profiles, lncRNA expression profiles and disease-associated genes to infer the potential associated diseases for human lncRNAs globally (Liu et al., 2014). In addition to the aforementioned datasets, Chen also incorporated the Gaussian interaction profile kernel similarity into their model and adopted the KATZ measure for lncRNA–disease association (Chen, 2015). Zhou et al. first constructed a heterogeneous network in terms of three sub-networks and then ranked the relevant lncRNAs for a given disease by applying the random walk with restart on the constructed network (Zhou et al., 2015). Chen et al. further improved the random walk with restart framework by initializing the probability vector according to the integration of lncRNA expression similarity and disease semantic similarity (Chen et al., 2016). Fu et al. decomposed the data matrices of heterogeneous data sources into low-rank matrices *via* matrix tri-factorization to explore the intrinsic as well as the shared structure, and then used the optimized low-rank matrices to obtain the potential associations (Fu et al., 2018). Lu et al. extracted a set of primary feature vectors and used the inductive matrix completion framework to infer the lncRNA-disease association (Lu et al., 2018). Lan et al. constructed a web server for lncRNA-disease association prediction by integrating multiple biological data resources (Lan et al., 2017). Xiao et al. obtained the association probability for a given lncRNA-disease association according to the lengths of the paths linking them in the constructed heterogeneous network (Xiao et al., 2018). Hu et al. adopted the bi-random walk algorithm to construct a linear model for the lncRNA-disease association prediction (Hu et al., 2019). Yu et al. applied a collaborative filtering model together with the Naive Bayesian Classifier on a constructed lncRNA-miRNA-disease tripartite network to effectively predict novel lncRNA-disease associations (Yu et al., 2019). Both Xie et al. and Chen et al. first fused different similarity matrices for lncRNAs and diseases based on a similarity kernel fusion model and then applied different classification frameworks to predict potential associations (Chen et al., 2019; Xie et al., 2019). Cui et al. developed a novel computational framework based on bipartite local model with nearest profile-based association inferring for prediction (Cui et al., 2019). Recently, Guo et al. employed the autoencoder to obtain the optimal feature space from the original feature set which was constructed from different types of similarities (Guo et al., 2019). The newly constructed features were then fed to a rotating forest to classify the lncRNA–disease associations and achieved remarkable performance.

Although the methods mentioned above have made great contributions to discover potential disease-related lncRNAs, the prediction accuracy is still limited in several ways. For example, in spite of the multiple biological data sources used in existing methods, the integration of the similarity matrices constructed from these data sources was simply performed by averaging them, which might be suboptimal. Furthermore, since the lncRNA–disease association data was relatively sparse, how to fully take advantage of the existing information during the prediction process remains challenging. To solve these issues, we here propose a multiview consensus graph learning method for disease-related lncRNAs prediction. Concretely, a set of similarity matrices for lncRNAs and diseases are first constructed by leveraging the known lncRNA–disease associations, respectively. We then iteratively learn a consensus graph from the multiple similarity matrices and obtain the final association probabilities between lncRNAs and diseases using a multi-label learning framework. To confirm the utility of our method, we compare the proposed method with several state-of-the-art methods on three widely used datasets under different evaluation metrics. The experimental results of various cross validation schemes clearly indicate that our method could achieve better prediction performance compared to the other three methods. Furthermore, we illustrate the potential of our method in identifying prognostic biomarkers for uterine cervical neoplasms in a case-study analysis.

## Materials and Methods

### Human lncRNA–Disease Associations

The LncRNADisease database is used as the data of known lncRNA–disease associations (Chen et al., 2013; Bao et al., 2019). We used three versions of LncRNADisease, June-2012 Version (marked as Dataset1), January-2014 Version (marked as Dataset2), and June-2015 Version (marked as Dataset3) in our experiments (Li et al., 2019). After filtering the lncRNA-disease associations with irregular disease names or lncRNA names and merging duplicate items, we obtained 276 interactions between 150 diseases and 112 lncRNAs for dataset1, 319 interactions between 169 diseases and 131 lncRNAs for dataset2, and 621 interactions between 226 diseases and 285 lncRNAs for dataset3, respectively (**Table 1**). For convenience, we use *Y* ∈ ℝ*^p^* ^×^ *^q^* to represent the known lncRNA–disease association matrix, where *p* and *q* denote the number of lncRNAs and diseases, respectively. If disease *j* has an association with lncRNA *i*, then *Y_ij_* = 1, otherwise *Y_ij_* = 0.

**Table 1:** Details of the three datasets used in this study.

Dataset	lncRNAs#	diseases#	interactions#
Dataset1	112	150	276
Dataset2	131	169	319
Dataset3	285	226	621

### Disease Semantic Similarity

To calculate the disease semantic similarity, we followed the same approach as described in previous work (Wang et al., 2010). Specifically, each disease *d* can be described by a Directed Acyclic Graphs (*DAG*s) that consists of three items *DAG* = (*d*, *T*(*d*), *E*(*d*)), where *T*(*d*) and *E*(*d*) are all the parent nodes of *d* including itself and all links from the ancestor nodes to child nodes, respectively. The contribution of disease *t* to the semantic value of disease *d* is defined as:

(1) $$\{\begin{array}{ll} D_{d}(t)=1 & \;if\;t=d\\ D_{d}(t)=\max\{0.5*D_{d}(t^{\prime })|t^{\prime }\in children\;of\;t\} & \;if\;t\neq d \end{array}$$

The overall semantic value of a given disease *d* can then be calculated as:

(2) $$D(d)=\sum_{t\in T(d)}D_{d}(t)$$

As a result, given a pair of diseases *i* and *j*, their semantic similarity is defined as:

(3) $$S(i,j)=\frac{\sum_{t\in T(i)\cap T(j)}\left(D_{i}(t)+D_{j}(t)\right)}{\sum_{t\in T(i)}D_{i}(t)+\sum_{t\in T(j)}D_{j}(t)}$$

We use *AD* ^(1)^ ∈ ℝ*^q^* ^×^ *^q^* to denote the obtained disease semantic similarity matrix and $AD_{ij}^{\left(1\right)}$ stands for the semantic similarity for a disease pair *i* and *j*.

### lncRNA Functional Similarity

Similarly, the lncRNA functional similarity was also calculated according to previous studies (Wang et al., 2010; Liang et al., 2019). For each lncRNA pair, we measured their similarity as follows:

(4) $$\mathrm{LFS}(i,j)=\frac{\sum_{d\in D(l_{j})}S(d,D(l_{i}))+\sum_{d\in D(l_{i})}S(d,D(l_{j}))}{m+n}$$

(5) $$S(d,D(l_{i}))=\underset{d_{1}\in D(l_{i})}{\max}(S(d,d_{1}))$$

where *m* and *n* are the number of diseases related to lncRNA *l_i_* and *l_j_*, and D(*l*) represents the disease set related to lncRNA *l*. We use *AL* ^(1)^ ∈ ℝ*^p^* ^×^ *^p^* to denote the obtained lncRNA functional similarity matrix and $AL_{ij}^{\left(1\right)}$ stands for the functional similarity for a pair of lncRNAs *i* and *j*.

### Gaussian Interaction Profile Kernel Similarity

Gaussian interaction profile kernel similarity is widely used in various semi-supervised prediction tasks for measuring similarities (Zou et al., 2016; Zhang et al., 2017; Zhu et al., 2018; Pan et al., 2019; Yin et al., 2019). Here we also adopted this similarity measure to construct the similarity matrices for lncRNAs and diseases, respectively. Concretely, given two lncRNAs *l_i_* and *l_j_*, their Gaussian interaction profile kernel similarity is defined as:

(6) $$\mathrm{KL}(l_{i},l_{j})=\exp(-\beta_{l}{\left\|\mathrm{IP}(l_{i})-IP(l_{j})\right\|}^{2})$$

(7) $$\beta_{l}=\beta_{l}^{\prime }/\left(\frac{1}{p}\sum_{i=1}^{p}{\left\|IP(l_{i})\right\|}^{2}\right)$$

where *IP*(*l_i_*) is in essence the *i*-th row of matrix *Y*, $\beta_{l}^{\prime }$ is a parameter controlling the kernel bandwidth and *p* is the number of lncRNAs. Similarly, for a pair of diseases *d_i_* and *d_j_*, we have:

(8) $$KD(d_{i},d_{j})=\exp(-\beta_{d}{\left\|IP(d_{i})-IP(d_{j})\right\|}^{2})$$

(9) $$\beta_{d}=\beta_{d}^{\prime }/\left(\frac{1}{q}\sum_{i=1}^{q}{\left\|\mathrm{IP}(d_{i})\right\|}^{2}\right)$$

where *IP*(*d_i_*) is in essence the *i*-th column of matrix *Y*, $\beta_{d}^{\prime }$ controls the kernel bandwidth and *q* is the number of diseases. Finally, we use *AD* ^(2)^ ∈ ℝ*^q^* ^×^ *^q^* and *AL* ^(2)^ ∈ ℝ*^p^* ^×^ *^p^* to denote the kernel similarity matrices for diseases and lncRNAs, respectively.

### Cosine Similarity

Cosine similarity is another effective method for measuring similarities and is widely used in recommender systems (Gao et al., 2019b; Zhang et al., 2019a). Therefore, we also adopted cosine similarity to build the similarity matrices for lncRNAs and diseases. The cosine similarity for a pair of lncRNAs or diseases is calculated as:

(10) $$\mathrm{CL}(l_{i},l_{j})=\frac{\mathrm{IP}(l_{i})\cdot \mathrm{IP}(l_{j})}{\left\|\mathrm{IP}(l_{i})\right\|\times \left\|\mathrm{IP}(l_{j})\right\|}$$

(11) $$\mathrm{CD}(d_{i},d_{j})=\frac{\mathrm{IP}(d_{i})\cdot \mathrm{IP}(d_{j})}{\left\|\mathrm{IP}(d_{i})\right\|\times \left\|\mathrm{IP}(d_{j})\right\|}$$

where the definition of *IP*(·)is the same as that in the previous section. As a result, we use *AD* ^(3)^ ∈ ℝ *^q^* ^×^ *^q^* and *AL* ^(3)^ ∈ ℝ*^p^* ^×^ *^p^* to record the cosine similarities for disease pairs and lncRNA pairs, respectively.

## Methods

### Notations

We first briefly introduce the notations used throughout the paper. All the matrices are denoted by italic uppercase letters while vectors are expressed in bold lowercase letters. The transpose, the trace and the Frobenius norm of a given matrix *M* are denoted by *M^T^*, *Tr*(*M*) and ||*M*||*_F_*, respectively. *M_ij_* represents the element at the *i*-th row and *j*-th column of *M*. **1** is a column vector with all elements equal to 1. For a given similarity matrix *S*, its degree matrix *D_S_* is a diagonal matrix whose main diagonal entry is ∑*_j_* (*S_ij_* + *S_ji_*)/2, and its Laplacian matrix *L_S_* is defined as *L_S_* = *D_S_* − (*S^T^* + *S*)/2.

### Multiview Consensus Graph Learning for LncRNA–Disease Association Prediction

Given a set of similarity matrices for both lncRNAs and diseases, our aim is to find an optimal consensus graph based on these similarity matrices for subsequent prediction. Specifically, suppose we have *n* similarity matrices *AD* ^(1)^, *AD* ^(2)^,….,*AD* ^(^ *^n^* ^)^ ∈ ℝ*^q^* ^×^ *^q^* constructed for diseases, and *m* similarity matrices *AL* ^(1)^, *AL* ^(2)^,….,*AL* ^(^ *^m^* ^)^ ∈ ℝ*^p^* ^×^ *^p^* for lncRNAs, we propose to learn a consensus graph for the disease space and lncRNA space from multiple views by the following objective function respectively (Han et al., 2018; Wang et al., 2020):

(12) $$\underset{SD,w_{D}^{\left(v\right)},F}{\min}{\left\|SD-\sum_{v=1}^{n}w_{D}^{\left(v\right)}AD^{\left(v\right)}\right\|}_{F}^{2}+2\alpha Tr(FL_{SD}F^{T}),\;s.t.\sum_{j=1}^{q}SD_{ij}=1,SD_{ij}\geq 0,\sum_{v=1}^{n}w_{D}^{\left(v\right)}=1,w_{D}^{\left(v\right)}\geq 0$$

(13) $$\underset{\mathrm{SL},w_{L}^{\left(u\right)},F}{\min}{\left\|SL-\sum_{u=1}^{m}w_{L}^{\left(u\right)}AL^{\left(u\right)}\right\|}_{F}^{2}+2\beta \mathrm{Tr}(F^{T}L_{SL}F),\;s.t.\sum_{j=1}^{p}SL_{ij}=1,\;SL_{ij}\geq 0,\sum_{u=1}^{m}w_{L}^{\left(u\right)}=1,w_{L}^{\left(u\right)}\geq 0$$

The weight parameters $w_{L}={\left[w_{L}^{\left(1\right)},\ldots ,w_{L}^{\left(m\right)}\right]}^{T}$ and $w_{D}={\left[w_{D}^{\left(1\right)},\ldots ,w_{D}^{\left(n\right)}\right]}^{T}$ added for each view guarantee that the objective functions in Eq. (12) and (13) adaptively learn an optimal consensus graph in terms of the importance of each view (Liu et al., 2018b). Finally, we integrate the optimization process from two spaces into one framework with graph-based multi-label learning and obtain the final objective function as follows:

(14) $$\begin{array}{l} \underset{\mathrm{SD},w_{D}^{\left(v\right)},SL,w_{L}^{\left(u\right)},F}{\min}{\left\|\mathrm{SD}-\sum_{v=1}^{n}w_{D}^{\left(v\right)}AD^{\left(v\right)}\right\|}_{F}^{2}+2\alpha Tr(FL_{SD}F^{T})+{\left\|SL-\sum_{u=1}^{m}w_{L}^{\left(u\right)}AL^{\left(u\right)}\right\|}_{F}^{2}+2\beta Tr(F^{T}L_{SL}F)+{\left\|F-Y\right\|}_{F}^{2},\\ s.t.\sum_{j=1}^{q}SD_{ij}=1,\;SD_{ij}\geq 0,\sum_{j=1}^{p}SL_{ij}=1,\;SL_{ij}\geq 0,\sum_{v=1}^{n}w_{D}^{\left(v\right)}=1,w_{D}^{\left(v\right)}\geq 0,\sum_{u=1}^{m}w_{L}^{\left(u\right)}=1,w_{L}^{\left(u\right)}\geq 0,F\in {\mathbb{R}}^{p\times q} \end{array}$$

where *L_SD_* and *L_SL_* are the Laplacian matrices for the similarity matrices *SD* and *SL*, *SD_ij_* and *SL_ij_* denote the (*i*,*j*)-th elements in *SD* and *SL*, respectively. The constraints imposed on both *SD* and *SL* ensures that the learned similarities have explicit meanings. *Y* is the known binary lncRNA-disease association matrix defined above. Specifically, the objective proposed in Eq. (14) has two advantages in predicting lncRNA–disease associations. First of all, it incorporates multiple data resources to learn a reliable similarity matrix and could be well adapted to arbitrary number of input similarity matrices. Moreover, the predicted label matrix *F* and the learned consensus graph can collaboratively guide the learning process of each other and thus lead to better results (Zhang et al., 2018a). We propose an efficient method to solve Eq. (14) in the following subsection.

### Optimization

In this section, we derive an efficient algorithm to solve the objective function in Eq. (14) in an iterative manner.

i) Updating *SD* and *SL*. For clarity, we only give the derivation for solving *SD* and the optimization for *SL* can be obtained similarly. By fixing the other variables in the objective function, Eq. (14) degenerates to Eq. (12). It can be rewritten in the following form:

(15) $$\underset{\mathrm{SD}}{\min}\sum_{i,j=1}^{q}{\left\|{\mathrm{SD}}_{ij}-\sum_{v=1}^{n}w_{D}^{\left(v\right)}{\mathrm{AD}}_{ij}^{\left(v\right)}\right\|}_{F}^{2}+\alpha \sum_{i,j=1}^{q}{\left\|F_{i}-F_{j}\right\|}_{2}^{2}SD_{ij},s.t.\sum_{j=1}^{q}SD_{ij}=1,\;0\leq SD_{ij}\leq 1$$

Since different rows of *SD* are independent, we can then solve each row separately:

(16) $$\underset{SD}{\min}\sum_{j=1}^{q}{\left\|SD_{ij}-\sum_{v=1}^{n}w_{D}^{\left(v\right)}AD_{ij}^{\left(v\right)}\right\|}_{F}^{2}+\alpha \sum_{j=1}^{q}{\left\|F_{i}-F_{j}\right\|}_{2}^{2}SD_{ij},s.t.\sum_{j=1}^{q}SD_{ij}=1,\;0\leq SD_{ij}\leq 1$$

Denoting *h_i_* as a vector whose *j*-th element is $h_{ij}={\left\|F_{i}-F_{j}\right\|}_{2}^{2}$, Eq. (16) can then be converted to:

(17) $$\underset{SD_{i}}{\min}{\left\|SD_{i}+(\frac{\alpha }{2}h_{i}-\sum_{v=1}^{n}w_{D}^{\left(v\right)}AD_{i}^{\left(v\right)})\right\|}_{2}^{2},s.t.SD_{i}1=1,0\leq SD_{ij}\leq 1$$

Eq. (17) could be solved by an efficient iterative algorithm proposed in (Huang et al., 2015).

ii) Updating *w_D_* and *w_L_*. When *SD*, *SL*, *F* and *w_L_* are fixed, Eq. (14) becomes:

(18) $$\underset{w_{D}}{\min}{\left\|SD-\sum_{v=1}^{n}w_{D}^{\left(v\right)}AD^{\left(v\right)}\right\|}_{F}^{2},s.t.w_{D}^{\left(v\right)}\geq 0,\sum_{v=1}^{n}w_{D}^{\left(v\right)}=1$$

To solve Eq. (18), we first convert the target graph *SD* into a column vector $a\in {\mathbb{R}}^{q^{2}\times 1}$ by stacking its columns together. Similarly, we convert the multiple input similarity matrices *AD* ^(^ *^v^* ^)^(*v* = 1,2…,*n*) into a set of vectors $G^{\left(1\right)},G^{\left(2\right)},\ldots ,G^{\left(n\right)}\in {\mathbb{R}}^{q^{2}\times 1}$ and denote a matrix *G* as $G=\left[G^{\left(1\right)},G^{\left(2\right)},\ldots ,G^{\left(n\right)}\right]\in {\mathbb{R}}^{q^{2}\times n}$. Then Eq. (18) can be transformed into:

(19) $$\underset{w_{D}}{\min}{\left\|a-Gw_{D}\right\|}_{2}^{2},s.t.w_{D}^{\left(v\right)}\geq 0,\sum_{v=1}^{n}w_{D}^{\left(v\right)}=1$$

Eq. (19) can also be solved by the algorithm proposed in (Huang et al., 2015; Liu et al., 2018a). The optimization for *w_L_* could be derived in a similar way.

iii) Update *F*. By fixing the other variables, Eq. (14) is reduced to the following problem:

(20) $$\underset{F}{\min}\;2\alpha Tr(FL_{\mathrm{SD}}F^{T})+2\beta Tr(F^{T}L_{SL}F)+{\left\|F-Y\right\|}_{F}^{2},s.t.F\in {\mathbb{R}}^{p\times q}$$

Taking the derivative of Eq. (20) with respect to *F* and setting it to zero, we have:

(21) $$(2\beta L_{SL}+I)F+2\alpha FL_{SD}=Y$$

Eq. (21) could be solved easily as a Sylvester equation (Zha et al., 2009; Shi et al., 2018).

**Figure 1:**
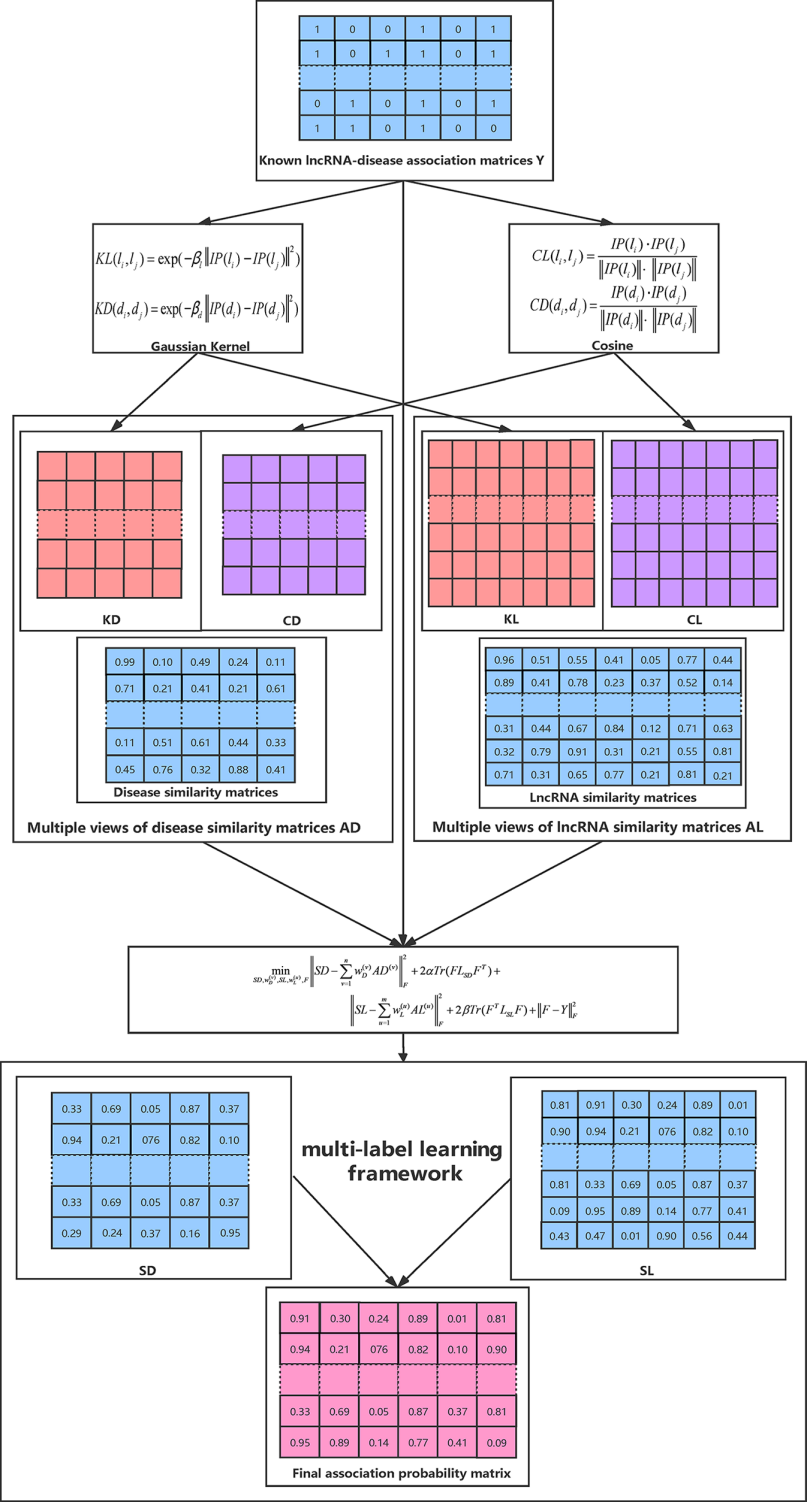
An overall workflow of our method.

The whole optimization process is summarized in Algorithm 1 and **Figure 1** illustrates the overall workflow of our method. Moreover, the source code of our method can be freely downloaded at: https://github.com/hjtan516/MCGLLDA.

**Algorithm 1:** 

**Input:** Known association matrix *Y* ∈ ℝ^*p* × *q*^, lncRNA similarity matrices {*AL* ^(1)^,*AL* ^(2)^,…,*AL* ^(m)^} from *m* views, disease similarity matrices {*AD* ^(1)^,*AD* ^(2)^,…,*AD* ^(n)^} from *n* views, parameters *α* and *β*. **Output:** Final association matrix *F*. 1. For each view of lncRNAs and diseases, initialize the weights as $w_{D}^{\left(v\right)}=1/n,w_{L}^{\left(u\right)}=1/m$; 2. While not converge do 3. While not converge do 4. Update *SD* according to Eq. (12); 5. Update *SL* according to Eq. (13); 6. Update *F* according to Eq. (21); 7. end while 8 Update $w_{D}^{\left(v\right)},w_{L}^{\left(u\right)}$ according to Eq. (19); 9. end while 10. return *F*

## Results

### Performance Evaluation

**Figure 2:**
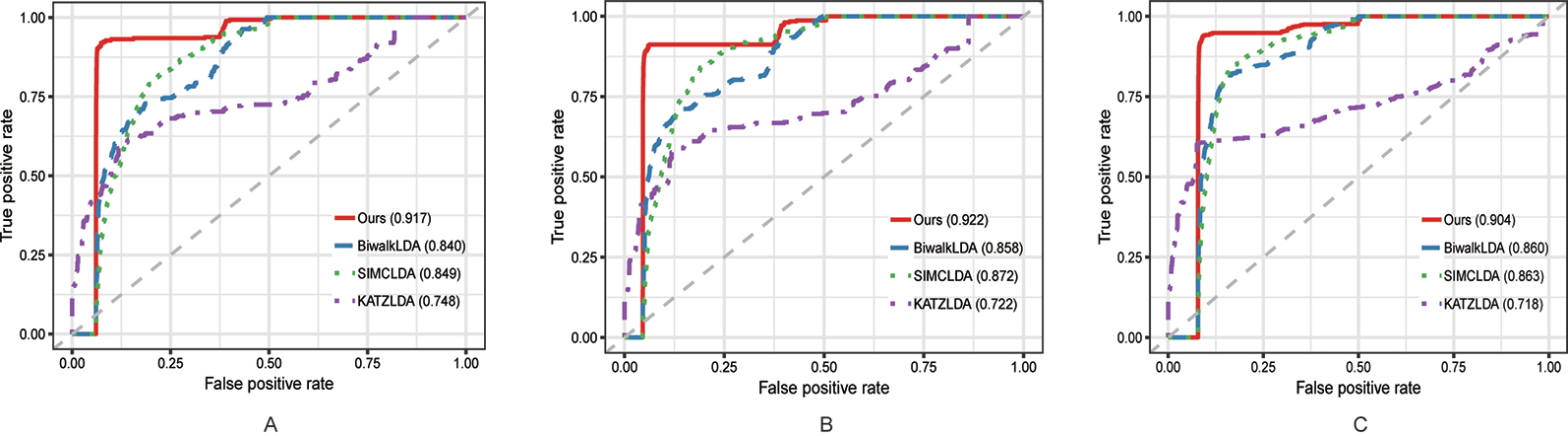
The comparison results between our method and the other three methods in terms of LOOCV using **(A)** Dataset1; **(B)** Dataset2; **(C)** Dataset3.

**Figure 3:**
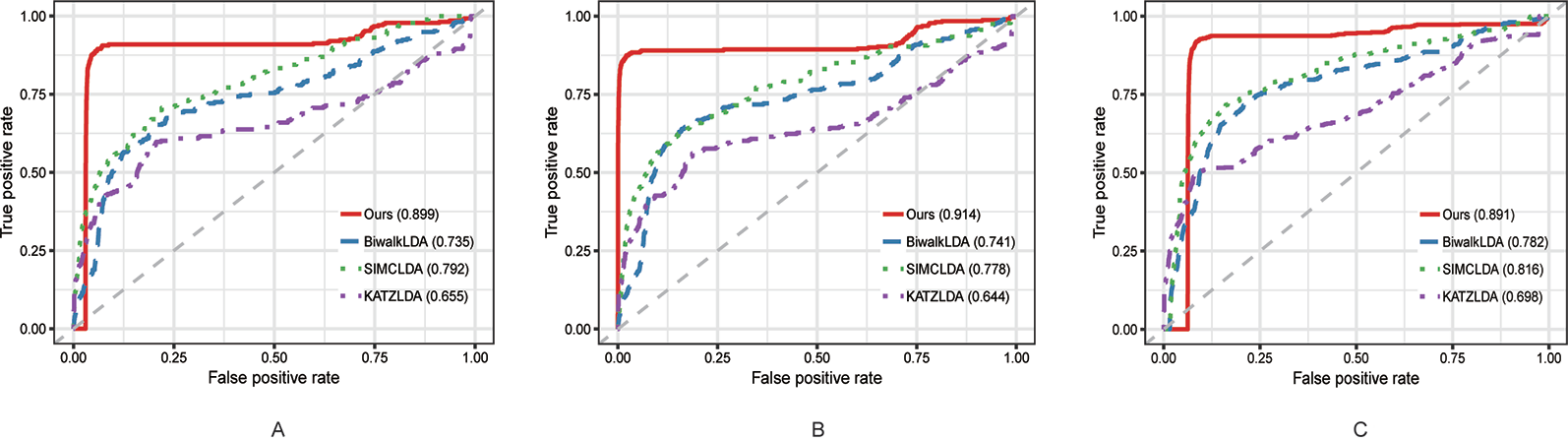
The comparison results between our method and the other three methods in terms of five-fold CV using **(A)** Dataset1; **(B)** Dataset2; **(C)** Dataset3.

In this section, we compared the proposed method with three state-of-the-art methods i.e. BiwalkLDA (Hu et al., 2019), SIMCLDA (Lu et al., 2018) and KATZLDA (Chen, 2015) on the aforementioned three datasets. Firstly, two evaluation metrics Leave-One-Out Cross Valuation (LOOCV) and five-fold Cross Validation (CV) were conducted to systematically evaluate the prediction performance of each method. Both LOOCV and five-fold CV take part of the known lncRNA–disease associations as test samples and use the remaining as the training samples. However, LOOCV only takes one association at a time as the test sample while in five-fold CV all the known associations are randomly divided into five parts and one part was used as the test set each time. The Receiver Operating Characteristic (ROC) Curve was plotted in terms of the cross validation results and the Area Under the ROC Curve (AUC) was calculated to measure the prediction accuracy. As shown in **Figures 2** and **3**, our method reached the highest AUCs on all three datasets in both LOOCV and five-fold CV.

**Figure 4:**
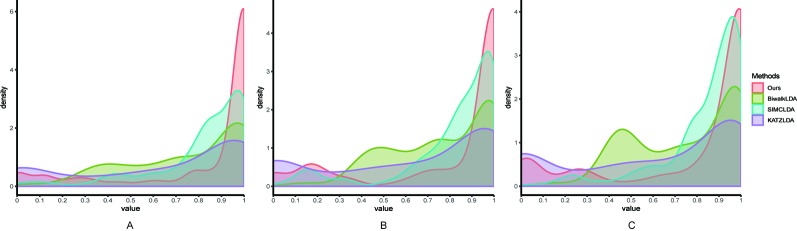
The comparison results between our method and the other three methods in terms of LODOCV using **(A)** Dataset1; **(B)** Dataset2; **(C)** Dataset3.

Next, we adopted Leave-One-Disease-Out Cross Validation (LODOCV) to test the ability of all methods in predicting the potential related lncRNAs for diseases without known associations. Specifically, for each disease, we removed all its associated lncRNAs and made predictions by leveraging the information from other diseases and lncRNAs. As a result, we could obtain a list of AUC values for each method and we used density plots to demonstrate the comparison results. As shown in **Figure 4**, compared with the other methods, our method obtained the highest numbers of AUC values greater than 0.9 on all three datasets. The Wilcoxon signed rank test also validated the significance of our method over the other three methods in terms of LODOCV (**Table 2**). In summary, these results clearly indicated that our method outperformed the other three methods in predicting reliable lncRNA–disease associations.

**Table 2:** Comparison of different methods based on LODOCV using Wilcoxon signed rank test.

Dataset	BiwalkLDA	SIMCLDA	KATZLDA
Dataset1	8.41e−10	1.84e−09	3.74e−12
Dataset2	4.57e–09	1.22e−07	8.07e−13
Dataset3	5.981e−09	7.49e−07	5.54e−14

### Parameter Analysis

**Figure 5:**
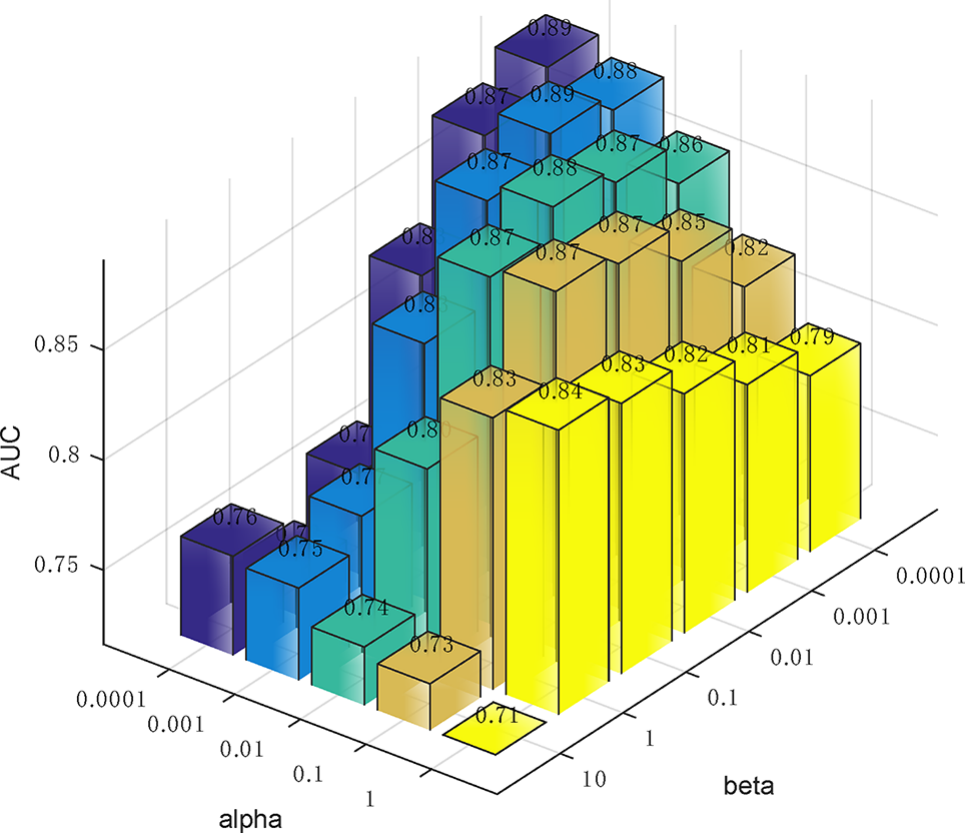
The influence of the two parameters *α* and *β* on the prediction accuracy of five-fold cross-validation.

In Eq. (14), we used two parameters *α* and *β* to balance the importance between the similarity graph learning and the predicted association matrix learning. We investigated the impacts of *α* and *β* on the prediction performance of our method. Specifically, *α* was tested in the range from 0.0001 to 1 and *β* was tested from 0.0001 to 10. To determine the best combination of *α* and *β*, five-fold cross validation was carried out on Dataset3. As a result, when both *α* and *β* were set to 0.0001, our method achieved the best performance (**Figure 5**).

### Convergence Analysis

**Figure 6:**
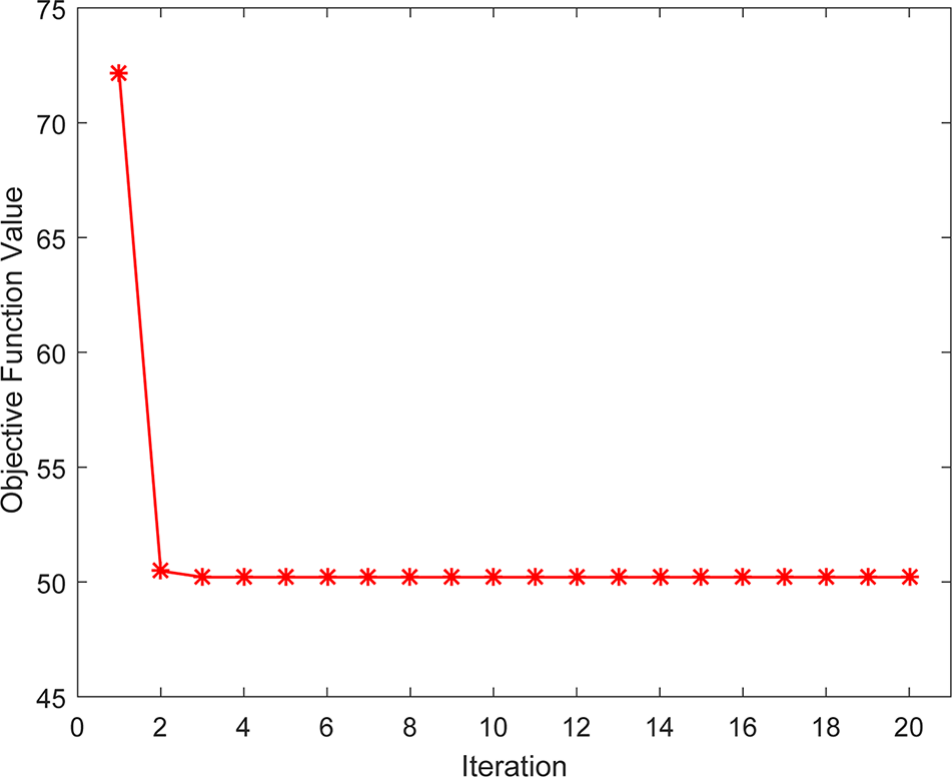
The convergence rate of our method.

We also studied the practical convergence speed of our method. Specifically, **Figure 6** illustrated the value variations of Eq. (14) with the number of iterations on Dataset3. As can be seen from the figure, the objective function value of Eq. (14) became stable in 5 iterations, indicating that our method converges rapidly and can be used in practice.

### Case Study

**Figure 7:**
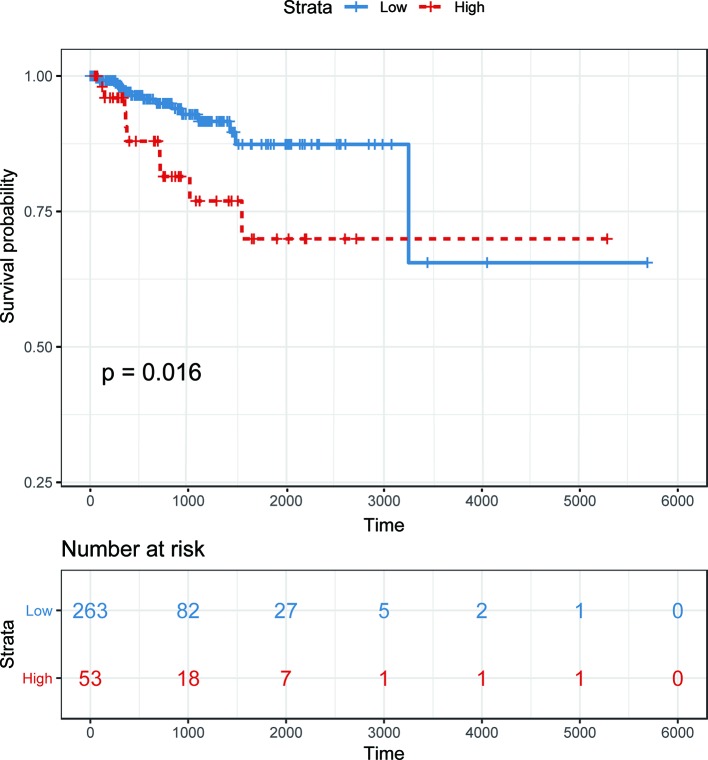
Kaplan–Meier survival analysis using MIR7-3HG as a prognostic biomarker in uterine cervical neoplasms. Patients are divided into “high” and “low” groups according to their expression level of MIR7-3HG against the mean expression level across all patients.

To demonstrate the potential of our method in identifying lncRNAs as meaningful biomarkers for a given disease, we carried out a case-study analysis on Uterine CErvical Neoplasms (UCEC). Uterine Cervical Neoplasms is one of the most frequent causes of death in women and its early detection can significantly decrease its death rate (Jeong et al., 2003). To make reliable predictions, we applied our method on a newer version (July-2017) of lncRNA–disease associations from LncRNADisease database. In particular, associations with lncRNAs that were not recorded in BioMart and diseases that were not included in the MeSH Category C for diseases were excluded during the implementation. The predicted associations were then validated by another two widely used databases recording disease-related lncRNAs, *i.e.* Lnc2Cancer (Ning et al., 2016) and MNDR (Wang et al., 2013). As expected, the two databases confirmed that 9 out of the top 10 predicted lncRNAs were verified to be related with UCEC (**Table 3**). The only unconfirmed lncRNA is MIR7-3HG. To evaluate whether this lncRNA might be involved in UCEC, we further downloaded the lncRNA expression profile of 316 UCEC samples from TANRIC (Li et al., 2015) and performed the Kaplan–Meier survival analysis by using MIR7-3HG as the biomarker accordingly (**Figure 7**). The statistical significance in the survival analysis was calculated using the log rank test (Bewick et al., 2004). Notably, the results demonstrated that the higher expression level of MIR7-3HG was related with significantly decreased survival rates of UCEC patients, indicating that MIR7-3HG might play an important role in the pathogenesis of UCEC.

**Table 3:** The top 10 predicted lncRNAs to be associated with cervical uterine neoplasms by our method.

Rank	lncRNA	Evidence
1	UCA1	Lnc2Cancer;MNDR
2	TUG1	Lnc2Cancer;MNDR
3	MIR99AHG	MNDR
4	MIR7-3HG	Unknown
5	HIF1A-AS1	MNDR
6	HOXC-AS1	MNDR
7	LINC-ROR	Lnc2Cancer
8	NEAT1	Lnc2Cancer;MNDR
9	GSEC	MNDR
10	HOTTIP	MNDR

## Conclusion

Increasing evidences have shown that lncRNAs accomplish a remarkable variety of biological functions and thus the aberrant expression or dysfunction of lncRNA might lead to various diseases. As a result, discovering newly disease-related lncRNAs might deepen our understanding of the biological roles of lncRNAs in carcinogenesis. In this work, a novel multiview consensus graph learning method for predicting lncRNA–disease associations was proposed. We first constructed a set of similarity matrices for lncRNAs and diseases by leveraging the known lncRNA–disease associations. We then learned a consensus graph for lncRNAs and diseases from the multiple similarity matrices and predicted the association probability between lncRNAs and diseases based on a multi-label learning framework. The results of LOOCV, five-fold CV as well as LODOCV on three widely used datasets all confirmed the superiority of our method. Moreover, the convergence analysis indicates that our method has a fast convergence rate and could be well adapted in practice. Lastly, the case study conducted for UCEC indicated that the expression level of MIR7-3HG was significantly related with the survival rate of patients and thus it might play important roles in the pathogenesis of UCEC. In summary, our method could reliably predict potential lncRNA–disease associations and could be easily extended to incorporate more data sources.

The success of our method is mainly two-fold. First, the known lncRNA–disease associations were leveraged to construct multiple kernel similarity matrices to better characterize the lncRNA similarities as well as disease similarities. Second, the view weights imposed for each view during the learning process guaranteed that more reliable similarity matrices have higher impacts on the final consensus graph. Despite the commendable results obtained, our method could still be improved in several ways. For example, the optimal values of the two parameters *α* and *β* might be searched by dynamic objective genetic algorithms. Besides, the integration of lncRNA expression data in our model should also be considered in the future.

## Data Availability Statement

The datasets generated for this study are available on request to the corresponding author.

## Author Contributions

CL conceived the study. HT and QS developed the algorithm and analyzed the results. CL and HT wrote the paper. CL and JL supervised the study. All authors have read and approved the final manuscript.

## Funding

This work was supported by the National Natural Science Foundation of China (Grant Nos. 61602283, 61873089, U1836216) and the Major Fundamental Research Project of Shandong Province (Grant No. ZR2019ZD03).

## Conflict of Interest

The authors declare that the research was conducted in the absence of any commercial or financial relationships that could be construed as a potential conflict of interest.
